# Tauroursodeoxycholic Acid Protects Retinal Ganglion Cells and Reduces Inflammation in Mice Following Optic Nerve Crush

**DOI:** 10.3390/ph18040569

**Published:** 2025-04-14

**Authors:** Nan Zhang, Ying Li, Xian Zhang, Micah A. Chrenek, Jiaxing Wang, Preston E. Girardot, Jana T. Sellers, Eldon E. Geisert, John M. Nickerson, Jeffrey H. Boatright

**Affiliations:** 1 Atlanta Veterans Administration Center for Visual and Cognitive Rehabilitation, Decatur, GA 30033, USA; nan.zhang@emory.edu (N.Z.); ying.li@emory.edu (Y.L.); xianz0707@csu.edu.cn (X.Z.); preston.girardot@emory.edu (P.E.G.); jana.t.sellers@emory.edu (J.T.S.); 2 Department of Ophthalmology, School of Medicine, Emory University, Atlanta, GA 30322, USA; micah.chrenek@emory.edu (M.A.C.); eldon.e.geisert@emory.edu (E.E.G.); jx.wang@med.miami.edu (J.W.); litjn@emory.edu (J.M.N.); 3 Department of Ophthalmology, Zhongnan Hospital, Wuhan University, Wuhan 430071, China; 4 Department of Ophthalmology, Second Xiangya Hospital of Central South University, Changsha 410011, China; 5 Bascom Palmer Eye Institute, University of Miami Miller School of Medicine, Miami, FL 33136, USA

**Keywords:** tauroursodeoxycholic acid, bile acid, retinal ganglion cell, inflammation, optic nerve crush, mus musculus, mouse, mice

## Abstract

**Purpose:** The aim of this study was to investigate the protective effects of systemically administered tauroursodeoxycholic acid (TUDCA) in an optic nerve crush (ONC) mouse model of retinal ganglion cell (RGC) death. **Methods:** C57BL/6J mice were injected intraperitoneally (i.p.) three times per week with TUDCA (500 mg/kg) for two weeks, after which unilateral ONC was performed. A control cohort was identically treated with a drug vehicle (phosphate buffered saline; PBS). A separate cohort did not undergo any injections or surgeries (this was termed the “Naïve” group). Pattern electroretinography (PERG) was recorded 3 days after ONC. Retinas were harvested for whole-mount immunofluorescence staining with an antibody against RGC marker Brn3a and imaged by fluorescent confocal microscopy. Apoptotic cells in the ganglion cell layer (GCL) were detected by Terminal Deoxynucleotidyl Transferase-Mediated dUTP Nick End Labeling (TUNEL) performed on fixed retina sections. Glial fibrillary acidic protein (GFAP) immunostaining on fixed retina sections was conducted to detect the activation of Müller cells. Total RNA was extracted from retinas and expression of interleukin (IL)-1β, IL-6, tumor necrosis factor (TNF)-α, and IL-10 was determined by digital droplet PCR (ddPCR). **Results:** TUDCA treatment preserved visual function as assessed by PERG. P1 and N2 amplitudes from the PBS-treated ONC group were significantly diminished compared to those of the Naïve group (*p* < 0.001). TUDCA treatment prevented this diminution. The amplitudes of P1 and N2 in the TUDCA-treated ONC group were statistically indistinguishable from those of the Naïve group and were higher than the PBS-treated ONC group (TUDCA+ONC vs. PBS+ONC, P1: 6.99 ± 0.89 µV vs. 3.60 ± 0.69 µV, *p* < 0.01; N2: −9.30 (IQR: −13.43–−6.44) µV vs. −4.47 (IQR: −10.26–−2.17) µV). TUDCA treatment preserved RGCs. The ONC-vehicle-only group had 25% fewer RGCs (Brn3a-positive cells) than Naïve eyes (*p* < 0.0001). TUDCA treatment nearly completely prevented this loss, preserving all but 7.7% of the RGCs, and the number of RGCs in the TUDCA-treated ONC group was significantly higher than in the PBS-treated ONC group (TUDCA+ONC vs. PBS+ONC, 1738.00 ± 14.43 cells per field vs. 1454.00 ± 6.55 cells per field, *p* < 0.0001). The number of TUNEL-positive cells in the GCL (Naïve vs. PBS+ONC group: 1.00 (IQR: 0.00–2.00) % vs. 37.00 (IQR: 8.50–48.50) %, *p* < 0.05) and GFAP-positive fibers transversing retina sections (Naïve vs. PBS+ONC group: 33.00 ± 1.15 vs. 185.70 ± 42.37 fibers/retina, *p* < 0.05), and the expression of IL-6, TNF-α were significantly greater in the PBS-treated ONC group compared to that of the Naïve group (Naïve vs. PBS+ONC group, IL-6: 0.07 (IQR: 0.06–0.31) vs. 0.99 (IQR: 0.56–1.47), *p* < 0.05, TNF-α: 0.19 ± 0.069 vs. 1.39 ± 0.23; *p* < 0.01), an increase not observed with TUDCA treatment. **Conclusions:** Systemic TUDCA treatment significantly preserved RGC function and survival in the mouse ONC model of RGC damage. TUDCA treatment prevented RGC apoptosis, Müller glial cell activation, and retinal expression of several inflammatory cytokines. These data suggest that TUDCA is a promising therapeutic candidate for preserving RGC numbers and function.

## 1. Introduction

Glaucoma blinds more than 75 million people worldwide [1]. It causes the death of retinal ganglion cells (RGCs), leading to visual field loss and blindness [2,3]. Elevated intraocular pressure is a significant risk factor for the disease [4]. However, even with well-managed intraocular pressure (IOP), RGC loss can progress, eventually resulting in vision loss [5]. As a chronic and progressive neurodegenerative disease, there is increasing interest in neuroprotective strategies and treatments that are independent of IOP management [6], including anti-glaucomatous agents, antibiotics, dietary supplements, novel small-molecule neuroprotectants, and neurotrophic factors [7]. However, as of now, no clinically recognized medication exists that effectively protects RGCs and the optic nerve in glaucoma treatment.

Optic nerve crush (ONC) is a surgical compression injury to the optic nerve, usually performed in rodents [8]. ONC causes axonal injury followed by retrograde degeneration and apoptosis of RGCs and has been widely used to model aspects of RGC damage [9,10,11]. Inflammatory retinal responses, including reactive gliosis and increased expression of pro-inflammatory cytokines after ONC, also contribute to RGC death and may mimic secondary RGC degeneration that occurs in glaucoma [12,13,14,15].

Tauroursodeoxycholic acid (TUDCA) is an endogenous bile acid and the taurine conjugate of ursodeoxycholic acid (UDCA) [16]. Recently, hydrophilic bile acids, notably TUDCA, have been reported to have profound anti-apoptotic and neuroprotective effects [17]. Systemic treatment with TUDCA is protective in models of diseases such as diabetes, kidney disease, Parkinson’s Disease, Huntington’s Disease, Alzheimer’s Disease, and retinal degeneration [18,19,20,21,22,23,24,25,26,27,28,29]. TUDCA has good bioavailability and is well tolerated when taken orally [30,31]. Further, clinical studies report that it is protective in neurodegenerative diseases [17], including increased survival in amyotrophic lateral sclerosis (ALS) patients [30]. Our previous studies reported that TUDCA offers protection in multiple retinal degeneration models, including diabetic retinopathy [32], retinitis pigmentosa (rd10 [33], and rd1 mice [34]). Additionally, TUDCA treatment is protective in animal models of RGC damage [35,36,37,38], though effects specifically on RGC function and in murine models are heretofore untested. The present study addressed this gap in knowledge.

Among several potential mechanisms of action [39,40], TUDCA has anti-inflammatory effects. It exhibits neuroprotective and anti-inflammatory effects in animal models of stroke by binding to the GPBAR1/TGR5 receptor in microglial cells [41]. It also reduces glial cell activation in multiple disease models including fatty liver disease, lipopolysaccharide-induced neuroinflammation, and retinitis pigmentosa [25,42,43,44], though this is relatively unexplored in models of RGC damage. Beyond its anti-inflammatory effects, extensive in vitro research on cultured neuronal cells and in vivo experiments on animal disease models have deeply explored and defined the anti-apoptotic properties of TUDCA [45]. TUDCA has been reported to prevent apoptosis by disrupting the E2F-1/p53/Bax apoptotic pathway and lowering nuclear fragmentation and caspase activity in Alzheimer’s disease [46]. TUDCA also prevents apoptosis by suppressing ER stress activation and oxidative stress on tunicamycin-induced apoptosis in primary cultured rat DRG neurons [47]. TUDCA reduced neuronal apoptosis and improved neurological functions after subarachnoid hemorrhage by targeting the TGR5/SIRT3 signaling pathway [48]. Based on the neuroprotective effects of TUDCA in neurodegenerative and retinal degenerative diseases, we hypothesize that TUDCA may exert potential neuroprotective effects on RGCs. Although a few studies have reported the protective effects of TUDCA in other animal models of RGC damage, they have not yet explored the impact on RGC function or the mechanisms underlying TUDCA’s protective action.

This study aimed to explore the protective effects of systemically administered TUDCA in a mouse model of RGC death induced by ONC, and whether TUDCA prevents or reduces RGC retinal inflammation. The rationale is a combination of the aforementioned need to develop IOP-independent treatments to protect RGC cells in glaucomatous disease, the oral bioavailability and tolerance in human subjects, the neuroprotective, anti-apoptotic, and anti-inflammatory properties we and others find with TUDCA treatment, including in ALS trials. We expanded our understanding of the protective effects that TUDCA treatment has on RGCs, including treatment effects on RGC function, apoptosis, and survival as assessed by retinal electrophysiology, TUNEL of retina section, and immunofluorescence labeling of RGCs in retinal flatmounts. Further, we assessed treatment effects on retinal inflammation via immunofluorescence staining of mouse retinal sections to observe Müller glia cell activation and digital drop PCR on retinal RNA to test for expression of pro-inflammatory genes. The results indicate that systemic TUDCA treatment significantly preserved RGC survival and function in an ONC mouse model of RGC damage. TUDCA treatment also significantly suppressed ONC-induced RGC apoptosis, activation of Müller glial cells, and overexpression of pro-inflammatory cytokines.

## 2. Results

### 2.1. TUDCA Treatment Preserves RGC Function After Optic Nerve Injury

**Figure 1:**
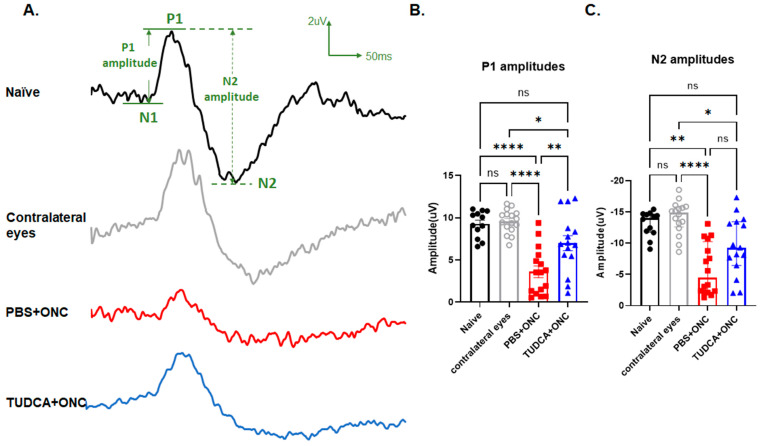
TUDCA treatment preserved RGC function 3 days after optic nerve crush. (**A**) Representative PERG waveforms from each group. (**B**) Quantification of P1 amplitudes 3 days after ONC. P1 amplitudes from PBS+ONC group were significantly diminished compared to those of the Naïve or Contralateral eye groups. TUDCA treatment preserved the P1 amplitudes significantly (*p* < 0.01). (**C**) Quantification of N2 amplitudes 3 days after ONC. N2 amplitudes from PBS-treated ONC group were significantly diminished compared to those of the Naïve group or Contralateral eye groups. TUDCA treatment preserved the N2 amplitudes significantly (*p* < 0.05). The results are represented as mean ± SEM (**B**), median ± IQR (**C**). A one-way ANOVA with Tukey’s multiple comparisons test (**B**) and Kruskal–Wallis test with Dunn‘s multiple comparison test (**C**) was conducted between the mean cells/field in all combinations. ns: no statistical difference, * *p* < 0.05, ** *p* < 0.01, **** *p* < 0.0001. n = 12 mice in Naïve group; n = 16 mice in PBS+ONC group and for the Contralateral eyes of that group; n = 15 mice in TUDCA + ONC group. PERG: pattern electroretinogram; RGC: retinal ganglion cell; PBS: phosphate buffered solution; ONC: optic nerve crush; TUDCA: tauroursodeoxycholic acid.

To test whether TUDCA can protect RGC function from injury, we performed PERG on mice 3 days after ONC. As shown in Figure 1, functional diminution was observed in eyes that underwent ONC in PBS-treated mice (“PBS+ONC”). These eyes exhibited markedly diminished P1 and N2 amplitudes compared with the contralateral eye that did not undergo ONC (62% decrease and 59% decrease separately, PBS+ONC vs. Contralateral, P1: 3.60 ± 0.70 µV vs. 9.59 ± 0.35 µV, *p* < 0.0001, one-way ANOVA with Tukey’s test; N2: −4.47 (IQR: −10.26–−2.17) µV vs. −14.89 (IQR: −15.82–−12.63) µV, *p* < 0.001, Kruskal–Wallis test with Dunn‘s multiple comparison test) or compared to eyes from Naïve mice (61% decrease and 55% decrease separately, PBS+ONC vs. Naïve, P1: 3.60 ± 0.70 µV vs. 9.27 ± 0.46 µV, *p* < 0.0001, one-way ANOVA with Tukey’s test; N2: −4.47 (IQR: −10.26–−2.17) µV vs.−14.05 (IQR: −14.68–−11.77) µV, *p* < 0.001, Kruskal–Wallis test with Dunn‘s multiple comparison test). Conversely, functional diminution was largely prevented in the eyes of TUDCA-treated mice that underwent ONC. TUDCA treatment preserved both P1 and N2 amplitudes compared with crushed eye from PBS-treated mice (P1: 6.99 ± 0.89 µV vs. 3.60 ± 0.69 µV, *p* < 0.01, one-way ANOVA with Tukey’s test; N2: −9.30 (IQR: −13.43–−6.44) µV vs. −4.47 (IQR: −10.26–−2.17) µV, Kruskal–Wallis test with Dunn‘s multiple comparison test). There was no statistically significant difference in PERG responses between the eyes of Naïve mice and the Contralateral eyes of PBS-treated mice or between the eyes of Naïve mice and ONC eyes of TUDCA-treated mice (Figure 1B,C), though functional preservation was not absolute.

### 2.2. TUDCA Treatment Maintains RGC Survival After Optic Nerve Injury

**Figure 2:**
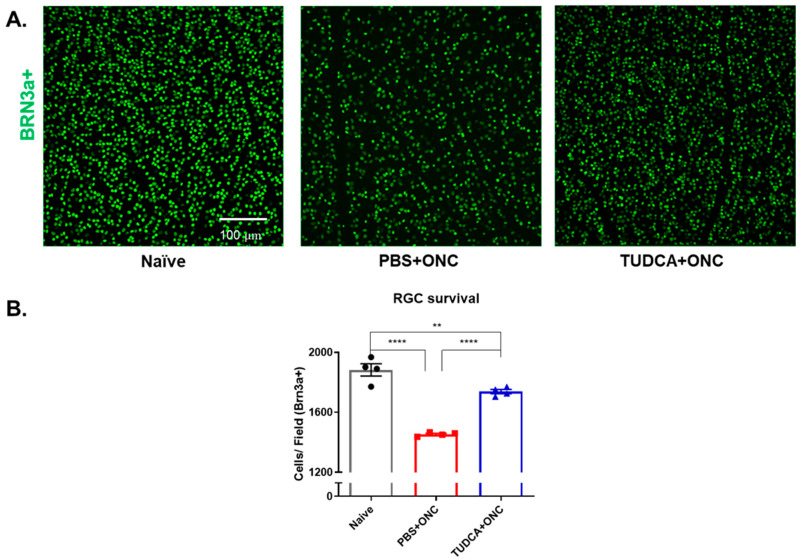
TUDCA treatment protects against ONC-induced RGC loss. (**A**) Representative images of retina flatmounts stained against Brn3a from different groups. (**B**) Quantification of Brn3a-positive cells, assumed to be RGCs, 3 days after ONC. The number of Brn3a-positive cells was significantly less in the PBS-treated ONC group 3 days after ONC compared to Naïve group (25% decrease). TUDCA treatment partially but significantly prevented this loss. The number of Brn3a-positive cells in TUDCA-treated ONC group was slightly lower than Naïve group (1883 ± 41.0 vs. 1738 ± 14.4 cells/field). The results are represented as mean ± SEM. A one-way ANOVA with Tukey’s multiple comparisons test was conducted between the mean cells/field in all combinations. ** *p* < 0.01, **** *p* < 0.0001. n = 4 mice per group. Scale bar = 100 microns. RGC: retinal ganglion cell; PBS: phosphate buffered solution; ONC: optic nerve crush; TUDCA: tauroursodeoxycholic acid.

RGC loss was observed within 3 days of ONC (Figure 2). As shown in Figure 2, the number of immunofluorescent Brn3a-positive cells, assumed to be RGCs, declined by about 25% in retinas from eyes that had undergone ONC in PBS-treated mice compared to retinas from Naïve mice (1454.00 ± 6.55 vs. 1883.00 ± 41.02 cells per field; n = 4/group, one-way ANOVA with Tukey’s test, *p* < 0.0001). Retinas from mice treated with TUDCA had significantly more RGCs compared to the PBS-treated mice after ONC (1738.00 ± 14.43 cells per field vs. 1454.00 ± 6.54 cells per field, n = 4, one-way ANOVA with Tukey’s test, *p* < 0.0001) (Figure 2B). TUDCA treatment did not perfectly preserve RGC numbers. The number of RGCs declined slightly but statistically significantly in retinas from eyes that had undergone ONC in TUDCA-treated mice compared to retinas from Naïve mice (1883.00 ± 41.02 vs. 1738.00 ± 14.43 cells per field; n = 4/group, one-way ANOVA with Tukey’s test, *p* < 0.01).

### 2.3. TUDCA Treatment Prevents ONC-Induced RGC Apoptosis

**Figure 3:**
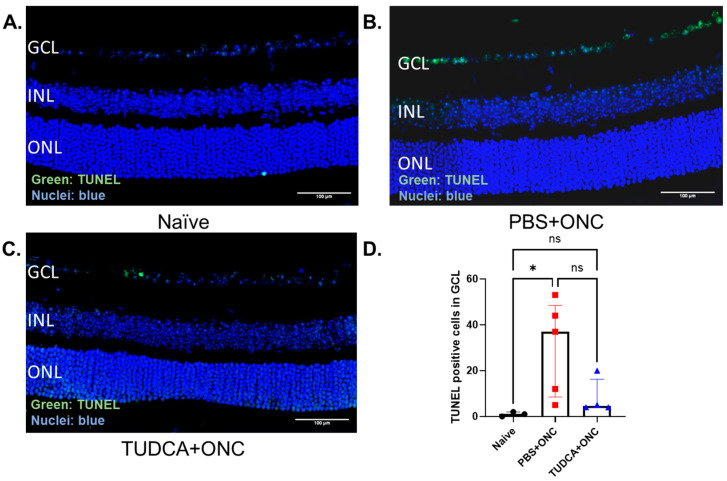
TUDCA treatment prevented ONC-induced RGC apoptosis. (**A**–**C**) Representative morphologic images of TUNEL staining from the region of 250–750 μm from the optic nerve. (**D**) Quantification of percentage of TUNEL positive RGC in GCL. RGC apoptosis significantly increased 3 days after ONC (*p* < 0.05). TUDCA treatment prevented the increase in TUNEL-positive cells of the GCL observed in retinas from eyes that had undergone ONC. TUNEL-positive cells in GCL were counted by masked observers across entire sections, which include optic nerve head. The results are represented as median ± IQR. Kruskal–Wallis test with Dunn‘s multiple comparison test was conducted between the mean percent in all combinations. ns: no statistical difference, * *p* < 0.05. n = 3 mice in Naïve group; n = 5 mice in PBS group; n = 4 mice in TUDCA group. Scale bar = 100 microns. GCL: ganglion cell layer; INL: inner nuclear layer; ONL: outer nuclear layer; TUNEL: terminal deoxynucleotidyl transferase dUTP nick end labeling; PBS: phosphate buffered solution; ONC: optic nerve crush; TUDCA: tauroursodeoxycholic acid.

TUNEL-positive cells in the GCL, presumed to be RGCs affected by axon crush [49,50], were observed and counted across entire retina sections. Compared with the Naïve group, which had few to no TUNEL-positive cells in the GCL (Figure 3A,D), the PBS-treated ONC group had a significant percentage of TUNEL-positive cells (Figure 3B,D) (Naïve vs. PBS+ONC group: 1.00 (IQR: 0.00–2.00) % vs. 37.00 (IQR: 8.50–48.50), *p* < 0.05, Kruskal–Wallis test with Dunn‘s multiple comparison test). In contrast, sections from the TUDCA-treated ONC group had few TUNEL-positive cells in the GCL (Figure 3C), an amount statistically indistinguishable from the Naïve group (Figure 3D).

### 2.4. TUDCA Treatment Prevents Müller Cell Activation After Optic Nerve Injury

**Figure 4:**
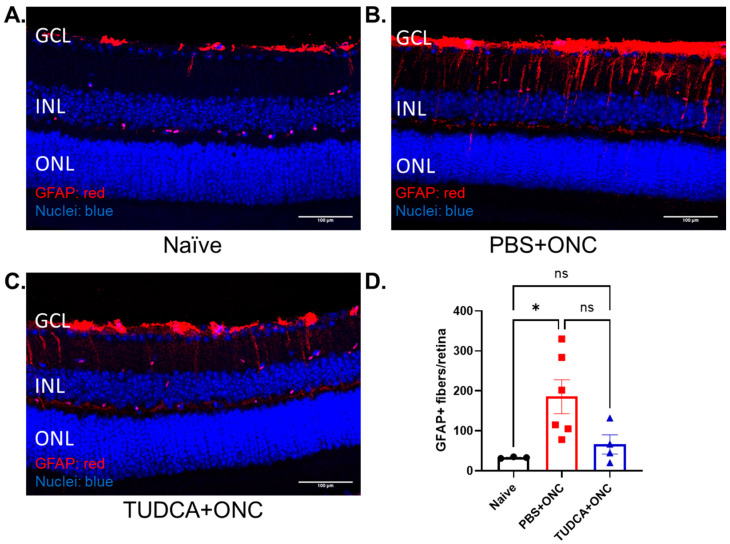
TUDCA treatment suppresses ONC-induced Müller cell activation. (**A**–**C**) Representative fluorescent images of GFAP staining from the region of 250–750 μm from the optic nerve. (**D**) Quantification of GFAP-positive fibers per retina. GFAP-positive fibers significantly increased 3 days after ONC. TUDCA treatment prevented the increase in number of GFAP-positive fibers in mouse retina caused by ONC damage. No significant difference was been found between Naïve and TUDCA treated groups. GFAP-positive fibers fully penetrating the INL were counted across entire sections by masked observers. The results are represented as mean ± SEM. A one-way ANOVA with Tukey’s multiple comparisons test was conducted between the mean cells/field in all combinations. ns: no statistical difference, * *p* < 0.05, ns: no significant difference. n = 3 mice in Naïve group; n = 6 mice in PBS group; n = 4 mice in TUDCA group. Scale bar = 100 microns. GCL: ganglion cell layer; INL: inner nuclear layer; ONL: outer nuclear layer; GFAP: glial fibrillary acidic protein; PBS: phosphate buffered solution; ONC: optic nerve crush; TUDCA: tauroursodeoxycholic acid. Control cohort “PBS+ONC” was shared across the experiment reported in Figure 4 in [55].

GFAP is widely used to assess the gliosis of Müller cells [51,52,53]. Here, we used retinal sections stained with GFAP to test whether TUDCA treatment might reduce Müller cell activation in our ONC model. The GFAP signal in Naïve retina sections was mainly in the inner retina (Figure 4A). In the ONC+PBS condition, the number of GFAP-positive cells increased, and the signal appeared to span the inner retina out to the outer plexiform layer (Figure 4B). Treatment with TUDCA prevented these ONC-induced changes in the GFAP signal (Figure 4C). The number of GFAP-positive fibers spanning from the ganglion cell layer (GCL) to the inner nuclear layer (INL) were counted by masked observers across entire sections for each cohort [54]; these quantified outcomes are presented in Figure 4D. ONC injury markedly increased the number of GFAP-positive fibers in PBS-treated mice compared with retinas from Naïve mice (Naïve vs. PBS+ONC group: 33.00 ± 1.5 vs. 185.70 ± 42.37 fibers/retina, n = 3 in Naïve group, n = 6 in PBS+ONC group, *p* < 0.05, one-way ANOVA with Tukey’s test), indicating that activation of Müller cells occurred after crush injury. TUDCA treatment prevented the Müller cell gliosis observed in retinas from eyes that had undergone ONC in PBS-treated mice, with the number of GFAP-positive strands being statistically indistinguishable between Naïve and TUDCA+ONC groups. These data suggest that ONC stressed the retina, leading to apparent Müller cell activation. TUDCA treatment prevented this stress and/or activation.

### 2.5. TUDCA Inhibits Inflammatory Response After Injury

**Figure 5:**
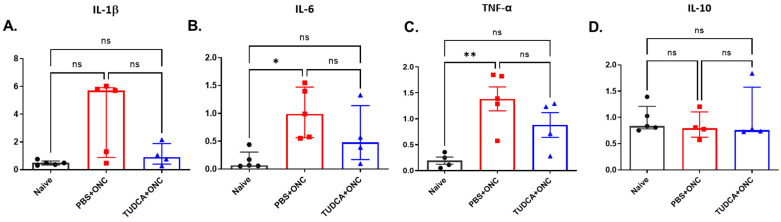
TUDCA treatment prevented ONC-induced overexpression of pro-inflammatory factors. The mRNA levels of IL-1β (**A**), IL-6 (**B**), TNF-α (**C**), IL-10 (**D**) were examined 3 days after ONC. *Y*-axis in all channels represent the mRNA ratio to HPRT multiplied by one thousand. The results are represented as mean ± SEM (**C**), median ± IQR (**A**,**B**,**D**). Kruskal–Wallis test with Dunn‘s multiple comparison test was conducted between the mean ratio to HPRT in all combinations (**A**,**B**,**D**). One-way ANOVA with Tukey’s tests was conducted between the mean ratio to HPRT in all combinations (**C**). ns: no statistical difference, * *p* < 0.05, ** *p* < 0.01, ns: no significant difference, n = 5 mice in Naïve group/PBS+ONC group; n = 4 mice in TUDCA+ONC group. IL-1β: interleukin 1β; IL-6: interleukin 6; IL-10: interleukin 10; TNF-α: tumor necrosis factor-α; PBS: phosphate buffered solution; ONC: optic nerve crush; TUDCA: tauroursodeoxycholic acid.

TUDCA treatment appears to suppress Müller cell activation induced by ONC (Figure 4). To further investigate whether TUDCA might reduce inflammation, we measured retinal mRNA expression levels of proinflammatory factors interleukin (IL)-1β, IL-6, and tumor necrosis factor-α (TNF-α). As shown in Figure 5, the mRNA expression levels of IL-6, and TNF-α were higher in the PBS-treated ONC group compared with the Naïve group (Naïve vs. PBS+ONC group, IL-6: 0.07 (IQR: 0.06–0.31) vs. 0.99 (IQR: 0.56–1.47), Kruskal–Wallis test with Dunn‘s multiple comparison test, *p* < 0.05, TNF-α: 0.19 ± 0.069 vs. 1.39 ± 0.23, one-way ANOVA with Tukey’s test, *p* < 0.01; n = 4–5 for each group; data reported as mRNA ratio to HPRT multiplied by 1000). There was no significant difference between Naïve and TUDCA-treated ONC groups in IL-6 and TNF-α mRNA levels, indicating that treatment with TUDCA prevented an ONC-induced increase in expression of these genes (Naïve vs. TUDCA+ONC group, IL-6: 0.07 (IQR: 0.06–0.31) vs.0.48 (IQR: 0.17–1.14), Kruskal–Wallis test with Dunn‘s multiple comparison test; TNF-α: 0.19 ± 0.069 vs. 0.88 ± 0.24, one-way ANOVA with Tukey’s test; n = 4–5 for each group; data reported as mRNA ratio to HPRT multiplied by 1000). Different from others, IL-1β and IL-10, did not show significant differences among groups (Figure 5D).

## 3. Discussion

In this study, we observed that TUDCA treatment significantly preserved RGC function enhanced RGC survival, and suppressed inflammation and apoptosis in an ONC mouse model of RGC damage. Prior to this, the protective effects of systemic TUDCA treatment on RGCs had not been tested in mice, nor had effects on RGC function or retinal inflammatory responses been assessed following ONC, regardless of species.

We found that systemic treatment with TUDCA partially preserved the number of RGCs in mouse eyes that had undergone ONC (Figure 2) and prevented apoptosis in the cells of the GCL (Figure 3). This confirms results in an ONC rat model [35] and extends findings into a new species. Further expanding on this, we found that systemic TUDCA treatment nearly completely protected against RGC functional loss caused by ONC, as shown by the preservation of amplitudes of P1 and N2 elements in PERGs (Figure 1). These functional findings may have clinical relevance as it is known that changes in PERG amplitude occur before visual field loss in glaucoma patients, and this has been used as a sensitive biomarker for monitoring RGC function in both glaucoma patients and animal models of glaucoma [56].

We observed that Müller glia cell activation and elevated production of proinflammatory factors occurred after ONC, but that both were attenuated with TUDCA treatment (Figure 4 and Figure 5). Inflammation plays an important role in the pathogenesis of glaucoma [57,58] in both glaucoma patients and animal models [59,60]. Upregulation of proinflammatory cytokines is observed in multiple glaucoma models [12,61,62,63]. Müller cell gliosis, assessed by GFAP upregulation [52,54,64], that occurs after ONC and other retinal damage can result in the release of glutamate, cytokines, and other mediators of inflammation and RGC injury [54,65,66]. We speculate that TUDCA treatment might promote RGC survival by inhibiting Müller cell activation and proinflammatory cytokine production to reduce neuroinflammation. An additional explanation is that TUDCA treatment prevented ONC-induced RGC apoptosis (Figure 3) via cell-autologous mechanisms (e.g., suppressing ER stress [67]), which would preclude signals from RGC-invoking inflammatory responses [13]. ONC mouse model serves as an acute RGC damage model, mimicking both glaucoma and acute optic nerve trauma.

The effects of systemic TUDCA treatment we report here in protecting RGC density and function and in diminishing retinal inflammation compare well with recently reported effects of other neuroprotective agents in the ONC mouse model. For instance, i.p. injection of the RXR agonist, 9-cis-13,14-dihydroretinoic acid (9CDHRA) diminished RGC apoptosis and retinal inflammation but had little or no protective effect on function [68]. A combination of ocular topical and i.p. injection of brimonidine modestly preserved RGC numbers (13% loss across retina without treatment, 10% loss with treatment), but had minimal effect on retinal inflammation [69]. Intravitreal injection of Brain-Derived Neurotrophic Factor (BDNF) modestly preserved RGC density but did not affect visual function loss [70]. Conversely, i.p. injections of the NAD+ precursor nicotinamide riboside (NR) significantly protected against losses in RGC density and function and suppressed retinal inflammation [55]. Thus, compared to other RGC neuroprotectants actively being studied, systemic treatment with TUDCA appears to be a candidate treatment worth pursuing.

A limitation of this study lies in its focus solely on the short-term protective effects of TUDCA, specifically examining its protective efficacy only three days after ONC injury, without addressing the long-term protective effects of TUDCA. Off-target effects of TUDCA were not observed (e.g., no gross changes were observed in behavior or in mouse weights with treatment). Another potential limitation of this study is that the effects of anesthesia and other animal handling were directly compared to outcomes of naïve, PBS+ONC, or TUDCA-ONC groups in experiments assessing RGC function via PERG (Figure 1), but not in other experiments. However, in addition to data from that experiment, we previously reported no observable differences between such sham treatment (contralateral, uninjured eyes from mice that underwent ONC) and naïve groups [55] and so did not repeat that comparison in every instance of the present study. Lastly, this study did not investigate the molecular mechanisms underlying TUDCA’s anti-inflammatory properties and its inhibition of RGCs apoptosis. To further elucidate the neuroprotective mechanisms of TUDCA, we aimed to explore earlier time points to identify immediate early gene responses following injury. The mechanisms of RGC protection for TUDCA are subjects of our future research. In addition, the additive or synergistic therapeutic effects of TUDCA combined with other glaucoma treatment medications, such as IOP-lowering drugs and other neuroprotective agents, will also be explored in our future studies to prevent RGC damage in glaucoma effectively.

## 4. Materials and Methods

### 4.1. Experimental Design

**Figure 6:**
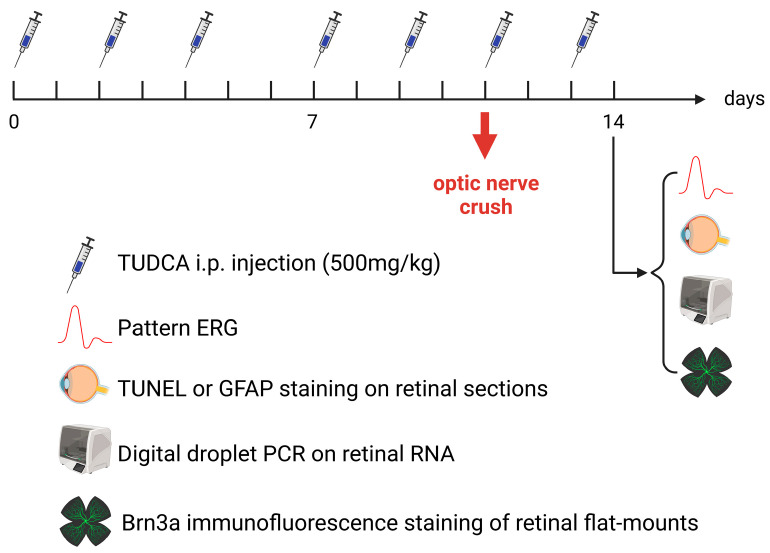
Experimental timeline. Details in text of Section 4.1.

Mice were divided into three groups: the Naïve group, the PBS+ONC group, and the TUDCA+ONC group. The Naïve group (12 mice) received no treatments. The PBS+ONC group (16 mice) received intraperitoneal (i.p.) injections of phosphate-buffered saline (PBS) and underwent unilateral optic nerve crush (ONC) surgery. The TUDCA + ONC group (15 mice) received i.p. injections of tauroursodeoxycholic acid (TUDCA) followed by unilateral ONC surgery. The experimental timeline is illustrated in Figure 6. Injections of PBS or TUDCA were administered on Monday, Wednesday, and Friday between 9:00 and 10:00 AM for two weeks. ONC surgery was performed 30 to 45 min after the sixth injection of either PBS or TUDCA. A seventh injection was given two days after the surgery. One day later, which was three days after the ONC, pattern electroretinograms (ERGs) were conducted, followed by euthanasia and tissue collection.

### 4.2. Animals

C57BL/6J mice (2–3 months old during experiments, weighing 20–25 g) were obtained from Jackson Laboratory (Stock# 000664, Bar Harbor, ME, USA). We maintained them on a 12 h light–dark cycle (7 a.m. on and 7 p.m. off) with food and water ad libitum. Mice were euthanized with CO_2_ gas or with cardiac perfusion. Husbandry and experimental procedures followed an approved protocol (Emory Institutional Animal Care and Use Committee Protocol ID PROTO201800248) and the “ARVO Statement for the Use of Animals in Ophthalmic and Vision Research”.

### 4.3. Drugs

We prepared and injected TUDCA as previously detailed [32,33,71]. Paraphrasing briefly from our prior studies, we dissolved TUDCA (Lot# 3310670, MilliporeSigma, Billerica, MA, USA) in phosphate-buffered saline (PBS; VWRVK813, Cat#97063-660, VWR International, LLC, Radnor, PA, USA). The 1× solution has a composition of 137 mM NaCl, 2.7 mM KCl, and 9.5 mM phosphate buffer. We prepared sterile TUDCA (5% (*w*/*v*)) solution just before injection, and adjusted the pH to 7.4, as previously detailed [71]. We injected TUDCA solution (i.p., 500 mg/kg mouse body weight) into mice in the morning 3 times per week as per our previous studies [32,33]. We injected “Control mice” with an equivalent volume of sterile PBS based on body weight.

### 4.4. Optic Nerve Crush

The ONC procedure was performed unilaterally following protocols we previously developed [72]. During the initial three days after the surgery, they were checked for potential infection, bleeding, and muscle control issues. A group of age-matched C57BL/6J mice that had no surgeries served as the Naïve group.

### 4.5. Pattern ERG

Three days after ONC, mice were dark-adapted overnight before pattern electro-retinograms (PERG) were performed, as previously described [70]. Summarizing and paraphrasing this procedure briefly, once mice were anesthetized (as described in the last section), 0.5% proparacaine and 1% tropicamide eye drops (Akorn Inc., Lake Forest, IL, USA) were administered to reduce eye sensitivity and dilate the pupils. Mice were placed on a heating pad (37 °C) under a dim red light provided by the overhead lamp of the Celeris-Diagnosys system (Diagnosys, LLC, Lowell, MA, USA). Transient PERG responses were recorded using black and white vertical stimuli. The pattern stimulator was placed on the tested eye, with the flash stimulator for the contralateral eye acting as a reference electrode. Pattern stimuli of 50 cd·s/m^2^ were presented. Six hundred averaged signals, with cut-off filter frequencies of 1 to 300 Hz were recorded under scotopic conditions. The P1 value was defined as the N1 trough to the peak of P1. N2 was defined as the preceding P1 peak to the trough of N2 (similar to what is termed “pERG amplitude” [73] or “P1N2” [74]). Mice were euthanized after PERG measurement by IACUC-approved CO_2_ asphyxiation.

### 4.6. RGC Quantification

Retinal ganglion cells (RGCs) were quantified after immunostaining whole flatmounts of the retina for Brn3a immunofluorescence, as we previously detailed [72]. Briefly summarizing our protocol, mice were deeply anesthetized and perfused with 4% paraformaldehyde. Eyes were removed and fixed in 4% paraformaldehyde for one hour at room temperature. Dissected retinas were washed in PBS containing 1% Triton X-100 (Sigma-Aldrich, Cat. # 9002-93-1, St. Louis, MO, USA) and blocked with 10% normal donkey serum and 5% BSA. The retinas were then placed in the primary antibody, Brn3a (1:1000; Santa Cruz; SC-31984; Dallas, TX, USA), and incubated overnight at 4 °C. Following this, each retina was washed three times for 10 min in PBST (PBS with 0.1% Tween-20) and incubated overnight at 4 °C with the secondary antibody (1:1000, Alexa Fluor 488 AffiniPure Donkey Anti-Goat, Invitrogen, Cat# ABS82, Eugene, OR, USA). After another three washes with PBST, the retinas were flat-mounted with coverslips using Fluoromount-G (Southern Biotech, Cat. # 0100-01, Birmingham, AL, USA). A Nikon C1 confocal microscope (Nikon, Inc., Melville, NY, USA) was used to image the RGCs. The analysis of RGC survival was performed as previously reported [72]. For each retina, four fields, each measuring 636.5 μm × 636.5 μm and located 1.0 mm from the optic nerve, were randomly sampled from the middle regions of each retina under 20× magnification. The images were processed using a custom pipeline in CellProfiler v.6 [75] (https://cellprofiler.org/citations [accessed on 16 April 2021]) to automatically count the number of Brn3a-positive cells in a masked manner [76]. To evaluate RGC survival for each retina, the number of RGCs counted from the four regions was averaged.

### 4.7. Immunochemistry

Mice were euthanized with CO_2_ gas after PERG. Eyes were fixed in fixation solution (97% methanol, Cat#: BDH20291GLP, VWR International, LLC, USA; 3% acetic acid, Cat#: Fisher BP2401-500, Thermo Fisher, Waltham, MA, USA) at −80 °C for 4 days, dehydrated in a series of methanol and xylenes baths, embedded in paraffin, and sectioned through the sagittal plane on a microtome at thickness of 5 μm, with minor variation of the freeze-substitution method of Ying et al. [77,78].

Immunofluorescence was used to detect glial fibrillary acidic protein (anti-GFAP, #Z0334, Dako, CA, USA) to assess Müller cell reactivity as we have previously described [79]. To ensure consistent examination across animals, sections encompassing the optic nerve head and the central corneal were chosen. One section from the retina of each animal was selected for the subsequent immunofluorescence staining.

Nikon Ti2 inverted microscope with A1R-HD25 confocal scanner was used for the imaging of the slides immunostained for GFAP (Nikon Instruments Inc., Melville, NY, USA) using a 20× objective lens, resonance scanning with 2× zoom at 1024 × 1024 per field, processed using Nikon’s Denoise.ai algorithm, and tiled using NIS Elements. GFAP quantification was achieved by manually counting GFAP-positive fibers fully penetrating the inner nuclear layer (INL) across whole sections as others have reported [54]. Control cohort “PBS+ONC” was shared across the experiment reported in Figure 4 of [55].

### 4.8. TUNEL Assay

One section from the retina of each animal that traverses the optic nerve was used for TUNEL (Terminal deoxynucleotidyl transferase dUTP nick end labeling) staining. TUNEL assay was performed according to the protocol for the DeadEnd Fluorometric TUNEL Kit (Cat: #G3250, Promega, Fitchburg, WI, USA) as we previously described [78]. To ensure consistent examination across animals, sections encompassing the optic nerve head and the central corneal were chosen. One section from the retina of each animal was selected for the subsequent immunofluorescence staining. The slides stained for TUNEL were imaged using a Nikon Ti2 inverted microscope with an A1R-HD25 confocal scanner (Nikon Instruments Inc., Melville, NY, USA), as described immediately above. TUNEL-positive cells in the GCL were manually counted across whole sections for each retina using Adobe Photoshop CS6.

### 4.9. RNA Extraction and Digital Droplet PCR

The retinal gene expression of IL-1β, IL-6, TNF-α, and IL-10 were assayed relative to hypoxanthine-guanine phosphoribosyltransferase (HPRT) expression by digital droplet PCR (ddPCR) as previously described [80]. Mice were euthanized, and retinal tissues were collected and flash-frozen on dry ice. Total RNA was isolated from each individual retina. RNA extraction and cDNA synthesis were achieved by following the standard protocols of Qiagen RNeasy mini kit (Cat: # 74106, Qiagen, Mississauga, ON, Canada) and Qiagen QuantiNova RT kit (Cat: #205410, Qiagen, Mississauga, ON, Canada) respectively [81]. 6-fluorescein amidite (FAM) hydrolysis probe sets for ddPCR were ordered from Integrated DNA Technologies (Coralville, IA, USA) or Biorad (Hercules, CA, USA) or for each target and normalized to a hexachlorofluorescein (HEX) HPRT hydrolysis probe set (Integrated DNA Technologies). ddPCR was performed using 5 ng of cDNA and FAM hydrolysis probe sets for IL-1β (Mm.PT.58.41616450; Integrated DNA Technologies, IA, USA), IL-6 (dMmuCPE5095532; Biorad, CA, USA), TNF-α (Mm.PT.58.12575861; Integrated DNA Technologies) and IL-10 (dMmuCPE5094026; Bio-Rad, Hercules, CA, USA), and a HEX hydrolysis probe set for HPRT (Mm.PT.39a22214828; Integrated DNA Technologies, IA, USA) in Biorad ddPCR Supermix for Probes (no deoxyuridine triphosphate [dUTP]). The droplets were prepared using an Automated Droplet Generator model AutoDG (Biorad, CA, USA), plates were sealed with a PX1 plate sealer, PCR was performed using a C1000 Touch thermal cycler (Bio-Rad, Hercules, CA, USA) with deep well block, and read using a QX200 droplet reader (Bio-Rad, Hercules, CA, USA). Data were analyzed using Bio-Rad QuantaSoft software v1.7.4.0917 and Microsoft (Redmond, WA, USA) Excel 2016.

### 4.10. Masking and Statistical Analyses

Trained observers conducting assessments that required judgments were masked to treatment group identities. This included marking PERG peaks and nadirs and counting of Brn3a-positive cells in retina flatmounts and of TUNEL-positive cells and GFAP-positive fibers in fixed retina sections. Statistical analyses were conducted using Prism 8.4.2 Software (GraphPad Software Inc., La Jolla, CA, USA). A Shapiro–Wilk test was used to check the normality of the distribution of results. Data that passed this check were tested for significant differences in treatment outcomes by one-way ANOVA with Tukey’s post hoc testing; data that did not undergo a Kruskal–Wallis test with Dunn‘s multiple comparison testing. The statistical test used for each result is identified in the figure captions. Every possible comparison between the three groups was considered. For all analyses, results were considered statistically significant when *p* < 0.05. Data are presented as mean ± SEM or median ± IQR.

## 5. Conclusions

We demonstrated that systemic TUDCA treatment significantly preserved RGC survival and RGC function in an ONC mouse model of RGC damage. TUDCA treatment also significantly suppressed activation of Müller glial cells, overexpression of some pro-inflammatory cytokines, and RGC apoptosis. These data suggest that TUDCA is a promising therapeutic candidate for preserving RGC function and delaying vision loss in glaucoma patients. However, more preclinical and clinical studies are needed to confirm the neuroprotective potential of TUDCA in glaucoma.

## Data Availability

All data are contained within the submitted manuscript. The data presented in this study are available in this submission.
